# Optimization and Validation of a High Throughput UHPLC-MS/MS Method for Determination of the EU Regulated Lipophilic Marine Toxins and Occurrence in Fresh and Processed Shellfish

**DOI:** 10.3390/md20030173

**Published:** 2022-02-26

**Authors:** Teresa D’Amore, Sonia Lo Magro, Valeria Vita, Aurelia Di Taranto

**Affiliations:** Istituto Zooprofilattico Sperimentale della Puglia e della Basilicata, Via Manfredonia 20, 71121 Foggia, Italy; sonia.lomagro@izspb.it (S.L.M.); valeria.vita@izspb.it (V.V.); aurelia.ditaranto@izspb.it (A.D.T.)

**Keywords:** biotoxins, okadaic acid, yessotoxin, pectenotoxin, azaspiracid, UHPLC-MS/MS, SPE

## Abstract

Under the name of lipophilic marine toxins, there are included more than 1000 toxic secondary metabolites, produced by phytoplankton, with the common chemical property of lipophilicity. Due to toxicological effects and geographical distribution, in European legislation relevant compounds are regulated, and their determination is accomplished with the reference liquid chromatography-tandem mass spectrometry method. In this study a modified ultra-high performance liquid chromatography-tandem mass spectrometry (UHPLC-MS/MS) method has been developed for the identification and quantification of EU-regulated lipophilic toxins. The method optimization included a refinement of SPE-C18 clean-up, in order to reduce matrix interferences. Improved LC conditions and upgraded chromatographic ammonia-based gradient ensured the best separation of all analytes and, in particular, of the two structural isomers (OA and DTX2). Also, different MS parameters were tested, and confirmation criteria finally established. The validation studies confirmed that all parameters were satisfactory. The requirements for precision (RSD% < 11.8% for each compound), trueness (recoveries from 73 to 101%) and sensitivity (limits of quantification in the range 3–8 µg kg^−1^) were fulfilled. The matrix effect, ranging from −9 to 19%, allowed the use of a calibration curve in solvent (3–320 µg kg^−1^ in matrix) for quantification of real samples. Method relative uncertainty ranged from 12 to 20.3%. Additionally, a total of 1000 shellfish samples was analysed, providing a first preliminary surveillance study that may contribute to the knowledge of lipophilic marine toxins contamination. Increase in algae proliferation events and intoxication cases, EFSA suggestions for modification of maximum permitted levels and toxicity equivalency factors, and new studies of important toxic effects underline that implementation of reference methods still represents an important task for health and food safety laboratories.

## 1. Introduction

Secondary metabolites from marine organisms have raised an uninterrupted interest in numerous fields, including the development of new drugs or drug leads, ecology, marine biology, toxicology and food safety.

In food system and nutritional toxicology, the production of stable poisoning metabolites, marine biotoxins (MBTXs, also known as phycotoxins), by several harmful microalgae species, and their bioaccumulation in the food chain represent a health and economic concern [1,2,3,4]. In addition, climate and hydrographic changes, environmental pollution and intensive aquaculture practices are either contributing to the increase of algae spreading phenomena or else experiencing periods of massive growth, the so-called “harmful algal blooms” (HABs) and, in consequence, to the accumulation of MBTXs in seafood, both from aquaculture and wild fisheries [5,6,7]. In particular, although there were reported cases of intoxication of various filter-feeders, such as cetaceans, fish and aquatic birds, the phycotoxins have natural tropism for lipid-enriched and hepatopancreatic gland tissues of shellfish, mainly mussels, oysters, clams, cockles and scallops [8,9].

According to the chemical properties, MBTXs are classified as lipophilic and hydrophilic. Lipophilic marine toxins (LMTs) are divided into sub-groups, which include the okadaic acid (OA) group, the pectenotoxin (PTX) group, the yessotoxin (YTX) group, the azaspiracid (AZA) group, the brevetoxin (BTX) group, the ciguatoxin (CTX) group and the cyclic imine (CI) toxins [10,11]. More than 1000 metabolites correlated with LMTs have been identified; however, in fulfilment of the European Food Safety Authority (EFSA) reports and studies, the European Commission (EC) set the maximum permitted levels (MPLs) for four subgroups in shellfish. The EC and Member States established the relevant compounds and assigned a toxic equivalency factor (TEF) to them [1,12,13,14,15]. Additionally, they appointed sampling plans, classified the location and boundaries of the production and relaying areas of bivalve molluscs, and planned regulatory controls and monitoring programs, according to Regulations (EC) No 853/2004 and 627/2019 [16,17,18].

In Table 1, the four subgroups of LMTs, the regulated analogues and their chemical and toxicological properties are summed up. The MPLs for each subgroup and TEF for each analogue are also shown. Several studies on mechanisms of action, toxicokinetics and the harmful effects of these toxins were reported; however, it is still necessary to clarify different aspects on the toxicodynamics of these toxins [19,20,21,22,23]. According to their poisoning symptoms and frequency of co-occurrence, OA and PTX groups were globally defined as DSP (diarrhetic shellfish poisoning) toxins [19,24]. However, the EFSA Panel on Contaminants in the Food Chain underlined that they do not share the same mechanism of action, so should not be included in the same regulatory limit [14]. In 2004, after the Joint FAO/IOC/WHO (Food and Agriculture Organization of the United Nations/Intergovernmental Oceanographic Commission of UNESCO/World Health Organization) report, the deregulation of pectenotoxins was proposed. Recent studies showed no oral toxicity in mice dosed with the PTX analogue PTX2 at 5000 µg kg^−1^ [8,25,26]. Therefore, in the Commission Delegated Regulation (EU) No 1374/2021 amending the Regulation (EC) No 853/2004, the deregulation was confirmed from September 2021 with the motivation “that there are no reports of adverse effects in humans associated with Pectenotoxins (PTX) group toxins” [27]. DTX3 is the unified term for forms of OA, DTX1, and DTX2 acylated/esterified with saturated and unsaturated fatty acids [1,2,28].

In this context, this study presents a rapid, high throughput and sensitive ultra-high performance liquid chromatography (UHPLC) coupled with ESI—triple quadrupole (QqQ) tandem mass spectrometry (MS/MS) method. The extraction and the clean-up steps, using solid phase extraction (SPE), were refined to reduce the important matrix effect. An evaluation of signal enrichment/suppression for all toxins was performed. The final protocol optimization also permitted the usage of minimum sample weight and solvent volume. Moreover, an optimized basic gradient was proposed to ensure the best separation between the two structural isomers (OA and DTX2). A full validation study was carried out, according to the EURACHEM and ICH guides, Decision 2002/657/EC and Regulation (EU) No 625/2017. Moreover, the quality control criteria prescribed by the EU-Harmonised Standard Operating Procedure for the determination of Lipophilic marine biotoxins in molluscs by LC-MS/MS (EU-RLMB SOP) were carefully evaluated [29,30,31,32,33]. The method was successfully applied to fresh, frozen, cooked, canned, and ripened molluscs. Also, 1000 samples (mussels, oysters, cockles, clams, scallops and squids) from Italy and foreign countries were collected and analyzed in the last three years (2019–2021). The large number of samples and the data obtained provide a first preliminary surveillance study that may contribute to the knowledge of LMTs contamination.

## 2. Materials and Methods

### 2.1. Chemicals and Working Standard Solutions

Water, methanol (MeOH) and acetonitrile (MeCN) of LC-MS grade and MeOH of HPLC grade were purchased from Carlo Erba Reagents (Rodano, Italy). Ammonium hydroxide (NH_4_OH, 32%), sodium hydroxide (NaOH, ≥99%) and hydrochloric acid (HCl, 37%) were from Merck KGaA (Darmstadt, Germany). Ultrapure water (H_2_O 18 MΩ/cm; Milli-Q, Millipore, Sigma Aldrich, Steinheim, Germany) was used in the clean-up phase. Standard stock solutions of LMTs in MeOH (OA, 8.37 mg L^−1^; DTX1; 8.52 mg L^−1^; DTX2, 3.78 mg L^−1^; PTX2, 4.41 mg L^−1^; AZA1, 1.30 mg L^−1^; AZA2, 1.22 mg L^−1^; AZA3, 1.18 mg L^−1^; YTX, 4.92 mg L^−1^; hYTX, 5.79 mg L^−1^) and freeze-dried mussel Certified Reference Material for Multiple Marine toxins (CRM-FDMT1, 3 g) were purchased from the National Research Council Canada NRCC (Halifax, Canada). Standard stock solutions at concentration of 1000 µg L^−1^ were prepared by taking appropriate volumes of each stock solution, depending on its concentration, and diluting them in MeOH. These solutions were stored at −20 °C for a maximum period of six months. Working standards at concentration of 80, 60, 40, 20, 10, 2 µg L^−1^ were obtained by appropriate dilution in MeOH and stored at −20 °C for a maximum period of one week. OASIS^®^ HLB 6 mL, 200 mg (Waters, Milford, MA, USA) cartridges placed on an Alltech (Venafro, Italy) 12-port vacuum manifold were used in the clean-up phase.

### 2.2. LC-MS/MS Analysis

Chromatographic separation was performed on an UHPLC system, an Ultimate 3000 (Thermo Fisher Scientific, Waltham, MA, USA). Two columns were compared: Acquity BEH C_18_ (2.1 mm × 100 mm; 1.7 μm) and Waters X-Bridge C_18_ (2.1 mm × 50 mm, 2.5 μm) (Waters, Milford, MA-USA). The latter column, coupled with a pre-column Security Guard ULTRA cartridge UPLC C18 for 2.1 mm (Phenomenex, Torrance, CA, USA), gave the best results (see below). The column heater was kept at 40 °C, while the autosampler compartment temperature was maintained at 15 °C. An injection volume of 5 μL and a flow rate of 0.2 mL min^−1^ were set. Both mobile phase A (water) and mobile phase B (acetonitrile—water 90:10, *v*/*v*) contained 0.046% *v*/*v* of NH_4_OH (pH = 11). Measurement of pH of the eluent was made with a SympHony pH-meter from VWR International (West Chester, PA, USA) using a combined glass electrode. Different elution gradients were tested. The optimized elution gradient, started with 20% B, was maintained for 0.5 min, and then followed by a linear increase to 85% B at minute 4.5. Then B concentration increased to 98% in 1 min and this composition was kept for 3 min. B concentration was finally lowered to 20% in 0.5 min, followed by 5 min of column re-equilibration. The total duration of the instrumental method was 14 min.

The analytes were detected by a triple quadrupole mass spectrometer TSQ-Endura (Thermo Fisher Scientific, Waltham, MA, USA), equipped with a heated electrospray source (H-ESI II) operating in both positive (ESI+) and negative mode (ESI−). Nitrogen (purity 99.999%) was used as sheath gas and auxiliary gas, while Argon (Ar, purity 99.9999%, Sapio s.r.l., Monza, Italy) was used as collision gas. The optimized parameters were: capillary voltage (3500 V in ESI+ and 2700 V in ESI−), sheath gas flow rate (30 arbitrary units), auxiliary gas flow rate (10 arbitrary units), ion transfer tube temperature (270 °C), vaporizer temperature (240 °C) and collision gas pressure (2.5 mTorr). The collision energy (CE) and the RF lens voltage were optimized for all toxins by direct infusion. Each toxin, diluted in 50:50 mobile phase A/B at the concentration of 100 µg L^−1^, was infused by syringe at a flow rate of 10 μL min^−1^ for determination of polarity mode, precursor and product ions. To identify each toxin, two transitions between the precursor ion and the three most abundant product ions were chosen among those detected. Table 2 shows the optimized MS/MS parameters, qualitative ion pairs and the quantitative ion pair for each analyte. The quantification of each toxin was determined using the external calibration method. The ion pair with the highest relative intensity was selected for quantification purposes, while the second and the third more intense ion pairs were used as qualifier ions for identification. Due to the unavailability of standard solutions for 45-OH-YTX and 45-OH-hYTX, literature data were used for precursor and product ions, while CE and RF lens voltage values were equal to those optimized for YTX and hYTX. The calibration curve constructed for YTX and hYTX was used for the quantification of 45-OH-YTX and 45-OH-hYTX in real samples. The system was interfaced via network chromatographic software (Chromeleon Xpress, Thermo Fisher Scientific, Waltham, MA, USA) and spectrometer control software (TSQ Tune Software), to a personal computer for control of the instruments, data acquisition and processing. The chromatograms were registered using XCalibur^TM^ 3.3 software (Thermo Fisher Scientific, Waltham, MA, USA). TraceFinder^TM^ 5.0 (Thermo Fisher Scientific, Waltham, MA, USA) was used for data processing and quality check.

### 2.3. Sample Preparation

Whole shellfish, washed with fresh water to remove sand and foreign material, were removed from the shells, and then drained for 10 min in a sieve. A representative sample portion of approximately 100 g of pooled tissues was homogenized in a blender for 1 min at room temperature. A test portion of 1.00 ± 0.05 g homogenate was weighted into a centrifuge tube and 4.5 mL of MeOH were added. The sample was suspended in the extractant by vortexing for at least 1 min at 1500 rpm. It was then centrifuged (4000 rpm × 10 min) at 10 °C, and the supernatant was transferred into a 15 mL polypropylene tube. The extraction procedure was performed twice. The final volume was made up to 10 mL with MeOH.

Processed samples were treated as follows. For cooked/frozen or cooked/vacuum packed samples, deionized water was added (30% of the weighted sample) prior to homogenization. On the contrary, canned samples, which contained liquids, were homogenized including those liquids. Then the extraction was performed as described above.

#### SPE Clean-Up

For the SPE, 5 mL of the methanolic shellfish extract were diluted with 5 mL of ultrapure water and loaded on OASIS^®^ HLB cartridge which had been conditioned with 6 mL of MeOH and 6 mL of MeOH-water (50% *v*/*v*). No wash steps were performed. The cartridges were eluted with 2 mL of MeOH containing 3% of NH_4_OH. A volume of 1 mL of the purified and concentrated extract was transferred in borosilicate glass vials before LC/MS-MS analysis. The remaining 1 mL was transferred into a polypropylene tube and hydrolyzed for detection of DTX3 forms. Briefly, 125 µL of NaOH 2.5 M was added to the extract, then it was vortexed for 20 s and placed in a heated bath (80 °C, 40 min). After cooling, 125 µL of HCl 2.5 M were added to neutralize the extract that was finally transferred in a borosilicate glass vial.

### 2.4. Validation Study

As currently required by European rules for the official control methods (ISO 17025:2017; Regulation (EU) No 625/2017), method validation is an indispensable prerequisite to judge an analytical method “fit for purposes”. The optimized method was validated by an in-house validation model, in agreement with Commission Decision 2002/657/EC at MPLs established in the Regulations (EC) No 853/2004 [16,30,31,34]. The parameters evaluated for analytical method validation were linearity, limit of detection (LoD) and limit of quantification (LoQ), selectivity, accuracy, measurement uncertainty, ruggedness and matrix effect. The accuracy was assessed, following ISO 5725–2, as intermediate precision and trueness [35]. The usage of both spiked samples and CRM-FDMT1 allowed a further requirement check. Fortification of shellfish samples was done prior to extraction. The suitability of the analytical method was tested both in crude and in hydrolyzed extracts. In Table 3 the measurement method for the determination of validation parameters was described. Moreover, during the validation study the quality control criteria prescribed by the EU-RLMB SOP regarding peak resolution (*Rs*), LoQs and linearity were carefully evaluated [29]. The *Rs* between the two isomers *OA* and *DTX2* was calculated according to the following equation:

(1) Rs=(tR−DTX2 −tR−OA (wDTX2 +wOA )2)

where *t_R_* is the retention time and *W* is the peak width (both in min).

The calibration curve for every analytical set was performed before and after the analysis of the samples, checking the criteria of correlation coefficient (*R*^2^ > 0.98) and slope variation (<25%) between the initial and final calibration curve. Intra-batch retention time drift <3% was also verified. The requirements of the EU-RLMB SOP to validate the LoQs of the analytical method under 40 µg kg^−1^ for AZA1 and OA, 50 µg kg^−1^ for PTX2 and 60 µg kg^−1^ for YTX were fulfilled. Finally, the unequivocal identification of each toxin was ensured by comparing the spectra of relative intensities of product ions and establishing a maximum tolerance of ±35%. In fact, as suggested by Commission Decision 2002/657/EC, the relative intensities of the detected ions, forming the spectra, shall correspond to those of the calibration standard solutions.

### 2.5. Interlaboratory Comparison: Proficiency Test Round

The optimized UHPLC-MS/MS method was further tested by an external quality assessment (proficiency test, PT), as recommended in the Regulation ISO/IEC 17025:2017 [34]. The PT materials, supplied by the National Reference Laboratory for Marine Biotoxins (Cesenatico, Italy), in compliance with the article 94 of Regulation (EU) No 625/2017 and requirement of ISO/IEC 17043:2010, consisted of two shellfish samples: ((1) *Mytilus edulis* and (2) *Mytilus galloprovincialis*) [31,36]. The sample was analyzed for the identification/quantification of LMTs. There were 11 participants. The analysis was performed twice, and the results were calculated as the mean of two measurements. The outcome was evaluated as the Z-score, satisfactory if |z| ≤ 3 (z = (x − x_a_)/σ_pt_, where x is the participant’s reported result, x_a_ is the assigned value and σ_pt_ is the standard deviation for proficiency).

### 2.6. Software and Statistical Analysis

Statistical analysis was used for evaluating method linearity and matrix effect, as described above. Moreover, the data obtained at each level of fortification were compared by using one-way ANOVA (*p* < 0.05), both in terms of recovery percentage and and relative standard deviation (RSD%). This comparison is necessary for verifying the homoscedasticity of values obtained at different levels. For statistical analysis of shellfish samples, the software JASP (Version 0.16, 2021) was used. For the purpose of pointing out possible differences and correlations in sample groups > LoQ, (203 samples), analytical results < LoQ were imputed as the highest LoQ of each toxin. This substitution approach for treating left-censored data is commonly known as the “upper bound” [37].

### 2.7. Sample Collection

The developed UHPLC-MS/MS method was applied for the determination of LMTs in 1000 samples, divided as follows: *Mytilidae* family (762 *Mytilus Galloprovincialis*, 17 *Mytilus Edulis*), *Veneridae* family (92 *Venus Gallina,* 7 *Ruditapes Philippinarum*, 4 *Callista Chione,* 3 *Meretrix Lyrata*, 2 *Ruditapes Decussatus*, 1 *Venus Verrucosa*), *Ostreidae* family (84 *Crassostrea Gigas*, 10 *Ostrea Edulis*), *Cardiidae* family (17 *Cardium Edule*), *Loliginidae* family (1 *Loligo Vulgaris*). The analyses were carried out by the Istituto Zooprofilattico Sperimentale della Puglia e della Basilicata (IZS-PB) over three years (2019–2021) for official control purposes. The territorially competent authorities established the sampling programme and collected the samples in agreement with Regulation (EU) No 627/2019 [17]. Each sample intended for laboratory testing weighed about 1 kg. The samples were analyzed in duplicate and the concentration was calculated as the mean of two measurements.

## 3. Results and Discussion

### 3.1. Sample Preparation Optimization

The exhaustive extraction of LMTs was usually performed with MeOH, as suggested by the EU-RLMB SOP [29]. Lower LoDs may be achieved by further reduction of the solvent volume of the crude extract [38]. This approach involves two drawbacks: (1) the impossibility of a complete dissolution of the extract in a reduced volume of solvent and (2) precipitation phenomena that may occur during the storage of the extract. In addition, in matrices such as shellfish, rich in lipids, proteins or pigments, matrix effect can occur that may lead to suppression or enrichment of signal, and consequently to over- or under-estimation of concentrations [39]. For example, it was reported that OA signal was enhanced in matrix, while AZA1 signal was suppressed [38,40,41]. Therefore, an additional purification procedure was performed. SPE clean-up is also an effective tool for analyte concentration. OASIS HLB sorbent is a kind of porous copolymer composed of hydrophobic divinylbenzene and hydrophilic *N*-vinylpyrrolidone. These polymeric cartridges can be applied for a broad range of acidic, neutral and basic compounds and are suitable for the purification of molecules with very different chemical properties, such as biotoxins. This kind of sorbent showed better results than C18 sorbent material, especially for YTX and hYTX. In order to enhance the adsorption of LMTs on the OASIS HLB columns, it was necessary to dilute the methanolic extract with an amount of water higher than 35% [42]. These *et al.*, who tested various SPE sorbent materials, chose a total volume of 10 mL of the methanolic mussel extract diluted with 50% of water (5 mL of crude extract + 5 mL of water) for the cartridge loading [38].

**Figure 1:**
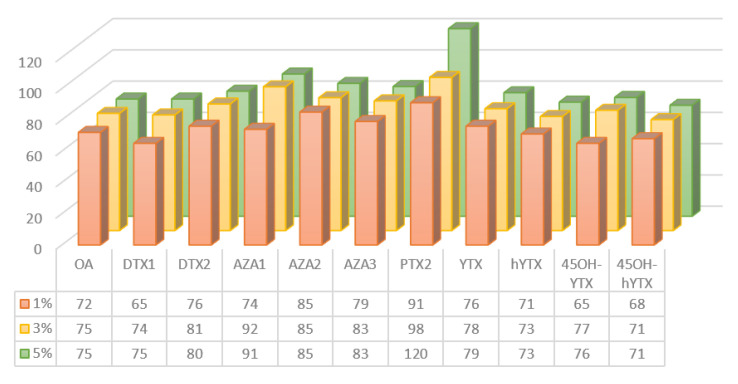
SPE clean-up optimization: comparison of three different methanolic ammonia elution solution (1%, 3%, 5% *v*/*v*).

According to literature data, MeOH was the best solvent in the elution phase of OA and PTX2, while for AZA1 MeOH with 1% of NH_4_OH is recommended [38,40,42,43]. The optimization of clean-up conditions required the comparison of three different elution solutions. The cartridges were eluted with 2 mL of MeOH containing three different concentrations of NH_4_OH (1%, 3%, 5% *v*/*v*). These tests were carried out using a mussel sample naturally contaminated with the YTX toxins group and spiked with other toxins at two concentrations (80 and 160 µg kg^−1^). In Figure 1 the different approaches tested for SPE clean-up are shown. A good compromise in terms of recovery (%) for all the LMTs was obtained with 3% NH_4_OH. These recovery values were further evaluated using the CRM-FDMT1, as described in Table 3.

### 3.2. Chromatographic Separation and Gradient Optimization

Since the LMTs contain functional groups, such as -SO_3_H, -COOH, -N=NH, they can be protonated or deprotonated depending on the pH of the solvent [44]. Hence, the retention time and the elution order of the toxins may be greatly affected by pH of the mobile phase, due to the charge state under different chromatographic conditions [45]. For chromatographic separation of LMTs, two approaches were described in the in EU-RLMB SOP:

(1) Acidic conditions, consisting in a mobile phase of water/acetonitrile with formic acid and ammonium formate. The chromatographic separation is obtained using columns with stationary phase C8 and C18 (BDS-Hypersil C8 (50 mm × 2 mm; 3 µm), X-Bridge C18 (50 mm × 2.1 mm; 2.5 micron)).

(2) Basic conditions, consisting in a mobile phase of water/acetonitrile with ammonia or ammonium bicarbonate. The chromatographic separation is obtained using cross-linked silica based C18 column materials stable up to pH 12. (X-Bridge C_18_ (150 mm × 2–3 mm, 3.5 μm).

In addition to various methods referred in the literature that used different mobile phases and chromatographic columns, under acid or basic conditions, an approach adopting a neutral pH gradient was described by Stobo et al. [46].

Preliminary experiments, carried out under acidic conditions described above, using Acquity BEH C18 and X-Bridge BEH C18 columns, gave asymmetric and broad peaks, in particular for YTXs.

**Figure 2:**
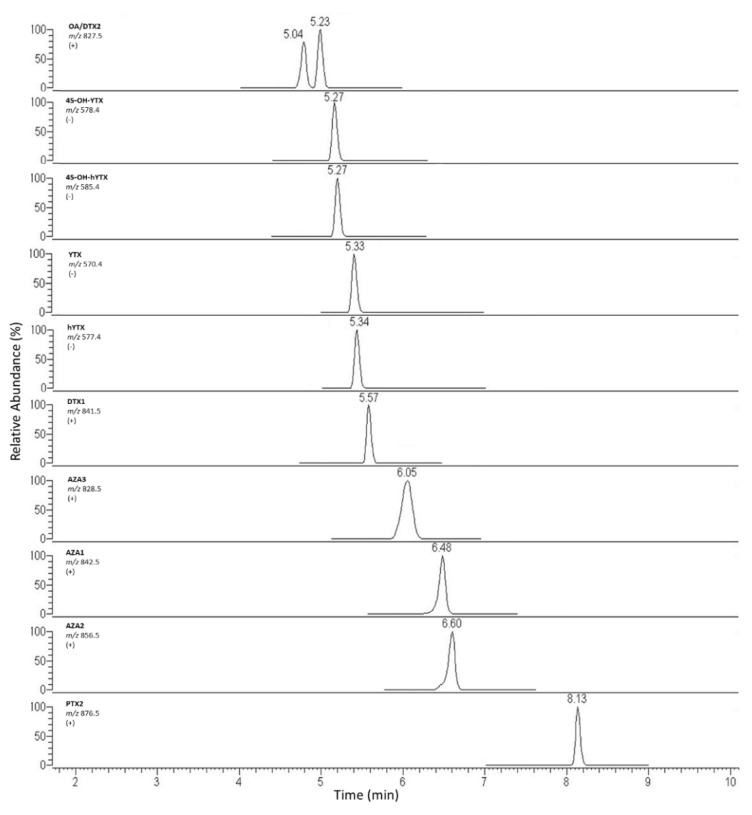
Chromatogram of mussel sample naturally contaminated with YTX toxins group, spiked with other toxins at a concentration of 160 µg kg^−1^.

The same columns were compared in basic conditions injecting a mix solution containing 9 LMT standards (OA, DTX2, YTX, hYTX, DTX1, AZA3, AZA1, AZA2, PTX2) at concentration of 20–40–80 µg kg^−1^. The best compromise between OA and DTX2 separation and column robustness and shelf-life in routine analyses was obtained using the X-Bridge column. Starting from mobile phase composition indicated by the EU-RLMB SOP for basic conditions, the elution gradient was optimized to achieve a *Rs* > 1 for OA and DTX2 [29]. In fact, a precise distinction between the two isomers appears a necessary condition, since the TEF values are different (1 and 0.6, respectively). Moreover, a recent technical report of the FAO/WHO Commission suggested lowering the TEF for DTX2 to 0.3–0.5, on the basis of oral LD50 studies [6]. Similarly, an excellent resolution, separation and symmetry for other toxins chromatographic peaks were achieved. The best analytical performances were obtained by using the following gradient: 20% B for 0.5 min, 85% B from 0.5 to 4.5 min, up to 98% in 1 min, maintained for 3 min, B concentration was finally lowered to 20% in 0.5 min, followed by 5 min of column re-equilibration. Flow rate: 0.200 mL min^−1^. Total run time: 14 min. Then this elution gradient was applied to spiked extract (20–40–80–160 µg kg^−1^) to assess possible peak shifting due to sample matrix interferences. Three replicates in three different working sessions were performed. The *t_R_* drift between standard and sample peaks was always <3%. In Figure 2, the chromatograms related to the injection of these samples are shown.

### 3.3. MS Parameters Optimization

#### 3.3.1. Acquisition Mode

The triple quadrupole mass analyzer offers various acquisition modes that can be adopted depending on the aim of the method. Regulations (EU) No 15/2011 and 627/2019 established that LC-MS/MS is the reference method for the official control of LMTs [17,47]. For this reason, selection of molecular ion (precursor) for fragmentation and analysis of the product ions is mandatory. The selection reaction monitoring (SRM) was chosen as the acquisition mode, using the product ion with the highest relative intensity for quantification purposes, and the second and the third most intense for identification. Therefore, adopting the identification points system reported in Commission Decision 2002/657/CE, monitoring both molecular ion and two product ions, 4 identification points were reached, resulting in an unequivocal identification for each toxin [30].

#### 3.3.2. SRM Method and Vaporization Temperature Optimization

For the optimization of ion source parameters, a single standard solution of each compound, prepared by dilution in mobile phase (50:50 *v*/*v* H_2_O and MeCN/H_2_O 90:10 both contained 0.046% of NH_4_OH *v*/*v*) at the concentration of 100 μg L^−1^, was infused at flow rate of 10 μL min^−1^ into the mass spectrometer. During method optimization, the modality of acquisition for each LMT was chosen, as well as other parameters, such as CE, RF lens voltage and gas flow optimized both in ESI+ and in ESI− acquisition (see Table 2). Although in most of methods OA group toxins were detected in negative ion mode, the most sensitive ionization conditions were achieved in positive ion mode due to sodium adduct formation. In agreement with the literature, YTX group toxins formed multicharged ion adducts in negative ion mode [48,49,50]. A common vaporization temperature value for all compounds was defined by injecting a 10 μg L^−1^ standard mix of the 9 toxins and increasing the values of ESI source vaporization temperature from 0 to 350 °C. A vaporization temperature value of 240 °C was chosen as the best compromise for detection of all toxins. This optimized MS condition allowed for the achievement of better sensitivities for all toxins.

### 3.4. Method Validation

After the optimization of conditions, the analytical performances of the developed method were evaluated in terms of selectivity, linearity, LoDs and LoQs, accuracy (precision and trueness), measurement uncertainty, matrix effect and ruggedness. All parameters complied with European requirements taken as a reference during this study. Method linearity was checked by the Mandel test: the calibration curves (range in matrix 3–320 µg L^−1^) gave a R^2^ higher than 0.98 for the single curves and for the mean curve, for all the analytes. For the selectivity study, the absence of interfering peaks within the retention time window ±3% was verified in processed and fresh mussel samples. In Table 4, relevant validation parameters and the confirmation criteria are reported. The LoQ values ranging from 3 to 8 µg kg^−1^ fulfilled the EU-RLMB SOP requirements [29]. As concerns precision and recovery, the data obtained from experiments described in Table 3, were preliminarily processed by Shapiro-Wilk test to verify the distribution normality. Intermediate precision, expressed as RSD%, was <11.8% for each analyte. The recoveries, as with most of the previously developed methods, exceeded the range 80–110%, so in the routine analyses, correction factors were applied to the results of real samples. The matrix effect, expressed in percentage, was evaluated using a calibration graph method for each toxin as shown in Supplementary Table S1. A value of 0% indicates no matrix effect, while values of <0% and >0% indicate ionization suppression and enhancement, respectively. Since matrix effectranged from 9 to 19%, due to the SPE clean-up step, the standard calibration curve in the solvent was used for real samples analysis.

The maximum measurement uncertainty, expressed in percentage, ranged from 12 to 20.3%.

In the ruggedness studies, matrix changes (major changes) were assessed. Blank samples of oysters, cockles, clams, scallops and squids were selected and spiked at 80 μg kg^−1^. The data obtained, expressed in terms of RSD% and recovery, were compared with the results reported for the validation matrix by means of Cochran and ANOVA tests. The results, reported in Table 4, indicate that it was possible to extend the application field of this method to these matrices. Furthermore, cooked mussels and clams were included among the matrices, since it is generally reported that the processing of shellfish (cooking, steaming, autoclaving) may lead to a considerable increase in the concentrations of LMTs. The extension of optimized methods to alternative matrices appears challenging, but very important. In recent times, in fact, it was indicated that LMTs, in particular OA group toxins, may bioaccumulate in marine biota and cause toxic effects in fish also [2,9].

### 3.5. Application to Naturally Contaminated Samples

LMTs concentrations in the 1000 mollusc samples are reported in Supplementary Table S2. According to EU legislation, the results were corrected, considering the TEFs [16]. Among them, 779 belong to the *Mytilidae* family, 109 to the *Veneridae* family, 94 to the *Ostreidae* family, 17 to the *Cardiidae* family, and one to the *Loliginidae* family. Of the 1000 samples, 203 (20.3%) had quantifiable values of the four groups of toxins (YTX, OA, PTX, AZA). A tentative statistical analysis, using one-way ANOVA and PCA, was made, but no significant correlation was found. Data of descriptive statistical analysis are shown in Table 5.

YTX toxins were quantifiable in 109 samples (10.9%), the most of which were collected in 2019. No sample was above the regulatory limits. A mean content of 105 µg kg^−1^ (range 30–1220 µg kg^−1^) was found. In 32 samples of mussels, YTX toxins co-occurred with OA toxins; in fact mussels were often exposed to a multi-toxin mixture [3]. Similarly, OA toxins were quantifiable in 126 samples (12.6%) with a mean content of 52 µg kg^−1^ (range 12–620 µg kg^−1^). Five samples (0.5%) were above the regulatory limits. In particular, four non-compliant samples were detected in April-May 2020, and all were *Mytilus Galloprovincialis*, while one was identified in September 2020 with the highest value (620 µg kg^−1^). In fact, OA toxins may increase, particularly in the spring and autumn seasons. However, algal bloom outbreaks still remain very difficult to predict due to the high variability of biotoxin content in phytoplankton cells [23,51].

The consultation of the EU RASFF (Rapid alert system for food and feed) portal, created “to ensure the flow of information to enabling swift reaction when risks to public health are detected in the food chain”, showed that another three notifications of OA toxins were reported in Italy during the period May-August in the category hazard “biotoxins”. In all three cases the risk decision was labelled as serious. In the last ten years (2010–2021), almost 100 notifications were reported (the most were from Italy, Spain, France, Ireland and Norway), and an increasing tendency seems to emerge, probably due to climatic and hydrographic changes.

PTX and AZA concentrations in all 1000 samples were <LoQ (8 µg kg^−1^), except in three and one samples, respectively. In the three PTX samples (two oysters and one mussels), OA toxins were also found, confirming the EFSA report suggesting the co-occurrence of these metabolites [1]. These data are in good agreement with previous literature, a similar trend of accumulation of YTX and OA toxins was reported by Schirone et al. and Visciano et al. in *Mytilus galloprovincialis* from the Adriatic Sea [11,18]. Moreover, the absence of AZA toxins in the Mediterranean Sea, except for rare cases, was also previously stated [51]. In fact, the unique sample above the LoQ for AZA toxins was a precooked frozen mussel (*Mytilus galloprovincialis*) that was imported.

### 3.6. Comparison with Other Methods

For official control purposes, the liquid chromatography tandem mass spectrometry (LC-MS/MS) method, proposed by the EU-RLMB SOP, was indicated as the reference method from 2014. During the last ten years, several LC-MS/MS and LC-HRMS methods were developed, and various modifications were advanced. An overview of these methods is summarized in Table 6. Due to the great amount of data, in this paper the attention is focused on the most recent and innovative procedures. Firstly, several extraction and clean-up procedures were extensively described in the literature. Apart from the classic methanolic extraction, complex matrices such as mussels required a following clean-up step, in order to reduce matrix interferences, improve selectivity and achieve LoDs. Solid phase extraction (SPE), liquid-liquid partition (LLP), and dispersive liquid–liquid microextraction (DLLME) are used to purify and concentrate the samples. Gerssen et al. demonstrated a significant reduction of the matrix effect by using SPE [52]. At the same time Regueiro et al. used online-SPE, ensuring high automation and the reduction of the matrix effect to < 5% [43]. Also, quick, easy, cheap, effective, rugged, and safe (QuEChERS) approaches, based on extraction with solvents/salts mixture followed by clean-up using dispersive solid-phase extraction (dSPE) with C18 sorbent, were investigated for sample preparation, since they allow for analysis of a wide range of matrices and analytes [48]. Wang et al. tested a modified QuEChERS extraction with methanol/ethanol/isopropanol combined with dSPE using graphene oxide as sorbent, obtaining a significant reduction of matrix interferences [39].

Liquid chromatography was used, and several approaches were proposed. Acidic, neutral or basic pH of mobile phases or usage of different columns, mainly C8 or C18, were extensively described. However, some drawbacks of these methods were described. Primarily, several chromatographic protocols did not guarantee a good separation of the two structural isomers, OA and DTX2. Recently, a method for detection of all EU-regulated marine toxins was presented, employing two different extractions for lipophilic and hydrophilic toxins and one chromatographic run. Despite good validation parameters, OA and DTX2 are detected together as one peak [53]. Similarly, Domenech et al. and Bosh-Orea et al. presented two high resolution methods for quantifying LMTs, but determined only OA and not its isomer [49,54]. Some protocols using QuEChERS with C18 or graphene-based sorbents, followed by hexane partition for removing the lipidic phase, were also described, but a good separation of OA/DTX2 was not achieved, and PTX toxins were not analyzed [39]. Also, Rubies et al. developed a QuEChERS protocol, prior to basic chromatographic separation and HRMS detection. Eprinomectin was used as the internal standard for controlling the matrix effect. Although it is reported that basic conditions ensure a better peak shape and, at the same time, the separation of all peaks, OA/DTX2 was partially overlapped [48]. On the contrary, in this work an optimized gradient ensured the best separation between these two isomers.

Finally, MS detection guarantees precise identification, selectivity and accurate quantification. Furthermore, it permitted, thanks to the untargeted analysis, the identification and the characterization of many new marine toxins and metabolites over the years. The usage of MS equipped with an electrospray ionization (ESI) source and a triple quadrupole (QqQ) analyzer is the most common also for routine analysis, due to its sensitivity and precise quantitation. Moreover, ESI-QqQ-MS/MS is certainly the most robust and performing detector [18,39,41,42,43,44,53,55,56]. Several methods using high resolution analyzers (Q-Orbitrap, Q-TRAP and Q-TOF) were described [38,41,45,48,49,50,54,57].

The improvement, the simplification and the optimization of the reference method remain important tasks for ensuring the accuracy, robustness and homogeneity of official data. Similarly, precision validation, considering not only “classical” parameters, but also the matrix effect, ruggedness and identification criteria, as proposed in this optimized analytical method, should be implemented.

### 3.7. Interlaboratory Comparison: Proficiency Test Round

The proficiency test results and evaluations are reported in Table 7. Among the 11 participants, only the IZS-PB laboratory performed SPE clean-up. All participants used MeOH as the extractant (double extraction). Only one other laboratory, apart from the IZS-PB, used a sample size of 1 g and a final solvent volume of 10 mL, while another one used 0.5 g and 5 mL. This is important, since the reduction of sample size and solvent waste represents some of the 12 main principles of green analytical chemistry [58]. The satisfactory Z-scores values confirmed the method reliability in analysis of LMT in mussels.

## 4. Conclusions

In this work, an optimized, sensitive and high throughput analytical method for the simultaneous determination of 11 lipophilic marine toxins and acylated/esterified forms in fresh and processed shellfish by ultra-high performance liquid chromatography coupled with tandem mass spectrometry was developed, refined for reducing solvent consumption, and validated. The sample preparation procedure consisted in double methanolic extraction followed by SPE clean-up that minimized the matrix effect. The ammonia-based gradient elution was highly optimized to ensure the best separation of the two isomers, OA/DTX2, and high selectivity. The method was fully validated in terms of linearity (*R*^2^ = 0.98), LoD (1–3 µg kg^−1^) and LoQ (3–8 µg kg^−1^), selectivity, precision (RSD = 11.8%), recovery (73–101%), measurement uncertainty (12–20.3%), matrix effect (−9–19%), and matrix ruggedness (oysters, cockles, clams, razor clams, scallops and cephalopod molluscs), in compliance with the most updated guidelines and regulations. The optimized method was applied for the analysis of 1000 commercial mollusc samples collected during the last three years (2019–2021). A preliminary monitoring study was also presented. Since algae proliferation events are increasing and new studies of important toxic effects of marine toxins are reported, the continuous surveillance and the implementation of analytical methods for determination of these toxicants appear to be mandatory.

## Figures and Tables

**Table 1:** Lipophilic marine toxin groups, relevant analogues, legal limits, chemical properties and toxicological information.

Biotoxin Group	Relevant and Regulated Compounds	Molecular Formula	Number of Identified Analogues	Legal Limit in Molluscs	TEF	Acute Reference Dose	Chemical Properties	Algae Producing Species	Mechanism of Toxicity	Main Symptomatology	Refs.
OA group	OA Okadaic acid	C_44_H_68_O_13_	>30	160 μg kg^−1^ of OA equivalents	1	0.3 μg eq. kg^−1^ b.w.	polyketide structure; C1 carboxyl group; polycyclic ethers and poly alkoxy with 3 spiro-keto rings; derivate from of a C38 fatty acid	*Dinophysis fortii, Dinophysis acuta, Dinophysis ovum, Dinophysis acuminate, Dinophysis norvegica,* *Prorocentrum lima Prorocentrum belizeanum, Phalacroma rotundata, Phalacroma mitra*	inhibition of serine/threonine protein phosphatases (PP) 2A, 1B, 2B	acute exposure: diarrhea, nausea, abdominal pain chronic exposure: mucosal damages of the intestinal tract, gastrointestinal cancer (tumor promoting, studies ongoing)	[1,2,3,6,7,8,9,11,19,23,24]
	DTX1 Dinophysistoxin-1	C_45_H_70_O_13_			1						
	DTX2 Dinophysistoxin-2	C_44_H_68_O_13_			0.6						
	DTX3 Dinophysistoxin-3	acylated derivatives of OA analogues (C length: C14–C22; most common is C16-palmitic) number of unsaturation: 0–6			1						
PTX group	PTX1 Pectenotoxin-1	C_47_H_70_O_15_	15	160 μg kg^−1^ of PTX equivalents	1	0.8 μg eq. kg^−1^ b.w.	macrolactonic structure; poly hydroxyl polycyclic ethers	*Dinophysis fortii, Dinophysis acuta, Dinophysis acuminate*	alteration of actin-based cytoskeleton, induction of apoptosis and subsequent cell death	liver necrosis, cardiac muscle damage (in vitro and in vivo: mice)	[2,3,6,8,11,14]
	PTX2 Pectenotoxin-2	C_47_H_70_O_14_			1						
AZA group	AZA1 Azaspiracid-1	C_47_H_71_NO_12_	>40	160 μg kg^−1^ of AZA equivalents	1	0.2 μg eq. kg^−1^ b.w.	poly hydroxyl polycyclic ethers; piperidine ring (amino group = aza group); C1 carboxyl group	*Azadinium spinosum, Amphidoma languida, Azadinium poporum*	cytotoxic effect by increasing of calcium and cAMP; alterations in cytoskeletal structures and the E-cadherin system, with disruption of cell-cell- and cell-matrix interactions, and perturbation of the intestinal barrier function	injury of lamina propria and epithelial cells in small intestine, liver and thymus necrosis (in vivo: mice)	[2,3,6,8,11,13,25]
	AZA2 Azaspiracid-2	C_48_H_73_NO_12_			1.8						
	AZA3 Azaspiracid-3	C_46_H_69_NO_12_			1.4						
YTX group	YTX Yessotoxin	C_55_H_82_O_21_S_2_	>90	3.75 mg kg^−1^ of YTX equivalents	1	25 μg eq. kg^−1^ b.w.	organosulfate structure (two sulfooxy groups); polycyclic ethers	*Protoceratium* *reticulatum*	modifications of intracellular levels of cAMP, calcium, PDEs, PKC and AKAP-149 (not well clarified)	immunotoxicity and immunosuppressive effects (in vitro and in vivo: rats)	[2,3,4,6,8,11,12,20,21,22]
	hYTX homoyessotoxin	C_56_H_84_O_21_S_2_			1						
	45-OH-YTX 45-hydroxy-yessotoxin	C_55_H_82_O_22_S_2_			1						
	45-OH-hYTX 45-hydroxy-homoyessotoxin	C_56_H_84_O_22_S_2_			0.5						

**Table 2:** Lipophilic marine toxins selected ion transitions (m/z), optimized collision energy and RF lens voltage values.

Compound	Ion	Polarity	Precursor Ion (*m*/*z*)	Product ion (*m*/*z*) ^1^ Quantifier	Collision Energy ^2^	Product Ion (*m*/*z*) ^1^ Qualifier	Collision Energy ^2^	RF Lens Voltage ^2^
OA	[M + Na]^+^	+	827.5	723.4	49	809.4 791.4	44 46	298
DTX2	[M + Na]^+^	+	827.5	723.4	49	809.4 791.4	44 46	218
YTX	[M − 2H]^2−^	-	570.4	467.2	30	502.2 386.2	23 31	298
hYTX	[M − 2H]^2−^	-	577.4	474.3	31	509.1 403.2	23 33	298
45-OH-YTX	[M − 2H]^2−^	-	578.4	467.4	31	396.4	31	298
45-OH-hYTX	[M − 2H]^2−^	-	585.4	474.0	31	403.4	31	298
DTX1	[M + Na]^+^	+	841.5	737.4	55	823.5 805.4	44 51	255
AZA3	[M + H]^+^	+	828.5	810.5	33	792.5 640.4	42 50	298
AZA1	[M + H]^+^	+	842.5	824.5	32	806.5 654.4	42 53	298
AZA2	[M + H]^+^	+	856.5	838.5	33	820.5 672.4	42 51	298
PTX2	[M + NH_4_]^+^	+	876.6	823.5	25	841.4 787.4	22 30	298

^1^ For each toxin, the quantifier and the two qualifier ions (first and second, respectively) are indicated, except for 45OH-YTX and 45OH-hYTX, for which only the quantifier and the first qualifier are reported. ^2^ Collision Energy and RF lens voltage values are expressed in V.

**Table 3:** Validation study.

Performance Characteristics	Evaluation/Measurement Approach
**Linearity**	Injection of LMTs standard solutions in methanol 2, 10, 20, 40, 60, 80 μg L^−1^ (three replicates at each concentration level) regression of calibration curve with the least square method. Mandel test to check linearity. Calculation of determination coefficient value (*R*^2^ > 0.98).
**Selectivity**	Analysis of 20 non-hydrolysed blank samples and 20 hydrolysed samples of fresh, frozen, precooked and canned mussels, for checking the absence of interfering peaks in the retention-time window of ± 3% of each analyte.
**Limit of detection** **Limit of quantification**	Gradual dilution (80, 40, 20, 10, 2, 1 μg L^−1^) of a matrix matched extract obtained by pooling the blank matrices used for selectivity study and spiking it with all the LMTs. The comparison of measured signals of quantifier ions with signals of blank samples, defined as signal-to-noise ratio (S/N), permitted the establishment of the minimum concentration at which the analyte could be reliably detected/quantified. A S/N of 3 and 10 for LoD and LoQ, respectively, was considered acceptable.
**Precision and trueness**	Analysis of a blank mussel sample spiked at 20 and 80 μg kg^−1^ with a mix LMTs standard solution (six replicates in two different working sessions with the same instrument, different days, operators and instrumental calibrations). The relative standard deviation for each analyte and recovery values were evaluated. Evaluation of method trueness by use of CRM-FDMT1: recovery values obtained on samples spiked at 80 μg kg^−1^ were used to correct the results of six independent tests obtained by using CRM-FDMT1.
**Measurement uncertainty**	Use of the maximum standard uncertainty approach: (2) Uf=(LoD2)2+(α×C)2 *Uf* is the maximum standard uncertainty (μg kg^−1^) *α* = numeric factor depending on the value of *C*.
**Matrix effect**	Evaluation using calibration graph method: as the ratio between the slope of the curve obtained for the matrix-matched extracts (matrix: mussels) and the slope of the curve for the standard calibration curve minus 1, expressed in percentage. (3) ME=(Slopematrix Slopesolvent−1)×100
**Matrix Ruggedness**	Conditions of major changes (matrix to analyze). Six additional experiments for each new matrix spiked at 80 μg kg^−1^ (oysters, clams, cockles, scallops and cephalopod molluscs). Comparison of precision and recovery data with the results obtained for validation matrix.

**Table 4:** Validation parameters.

Compound	LoQ µg kg^−1^	Precision (Mean) RSD%	Recovery (Mean) %	Identification Criteria (Ion Ratio % Qualifier 1/Quantifier) ± 35%	Matrix Effect %	Selectivity	Matrix RUGGEDNESS
OA	8	7.8	75.4	59 ± 35	−9	verified for fresh, frozen, precooked and canned mussels	oysters, cockles, clams, scallops and cephalopod molluscs ^(a,b)^
DTX2	7	8.2	81.6	59 ± 35	−6		
YTX	8	8.1	73.8	31 ± 35	−7		
hYTX	5	8.9	73.1	31 ± 35	−3		
45-OH-YTX	4	8.1	73.8	31 ± 35	/		
45-OH-hYTX	5	8.9	73.1	31 ± 35	/		
DTX1	7	8.2	76.4	54 ± 35	−2		
AZA3	8	3.6	82.3	23 ± 35	6		
AZA1	4	4.4	87.4	23 ± 35	−8		
AZA2	3	3.4	81.8	23 ± 35	11		
PTX2	3	11.8	101.3	71 ± 35	19		

^a^ Cochran test results: gobs = 0.369 < gcrit (0.95;24;4) = 0.393. ^b^ F-test results: Fobs = 2.873 < Fcrit (0.95) = 3.682.

**Table 5:** Statistical analysis and concentrations (µg kg^−1^) of lipophilic marine toxins in 203 shellfish samples above the limit of quantification grouped by toxin group, year and family.

	N	Median	Mean	SD ^1^	IQR ^2^	Min	Max	25th Percentile	75th Percentile
OA group	203	19.0	35.3	57.8	35.0	8.00	620	8.00	43.0
YTX group	203	30.0	60.0	109	62.0	8.00	1220	8.00	70.0
PTX group	203	8.00	8.19	1.65	0.00	8.00	27.0	8.00	8.00
AZA group	203	8.00	8.05	0.84	0.00	8.00	20.0	8.00	8.00
**OA Group per Year**									
2019	101	18.0	28.9	62.4	24.0	8.00	620	8.00	32.0
2020	77	31.0	50.9	57.1	40.0	8.00	278	17.0	57.0
2021	25	8.00	12.9	13.2	0.00	8.00	58.0	8.00	8.0
**YTX Group per Year**									
2019	101	40.0	72.0	144	66.0	8.00	1220	8.00	74.0
2020	77	8.00	36.0	50.0	42.0	8.00	260	8.00	50.0
2021	25	90.0	82.0	49.0	40.0	8.00	180	60.0	100
**OA Group per Family**									
*Mytilidae*	197	19.0	35.3	58.6	35.0	8.00	620	8.00	43.0
*Ostreidae*	6	30.0	35.9	19.19	24.8	18.0	66.0	30.0	46.5
**YTX Group per Family**									
*Mytilidae*	197	30.0	61.0	0.11	66.0	8.00	1220	8.00	74.0
*Ostreidae*	6	8.00	8.00	0.0	0.0	8.00	8.00	8.00	8.00

^1^ standard deviation; ^2^ interquartile range.

**Table 6:** Recent instrumental methods for the determination of lipophilic marine toxins.

References	Extraction and Clean-Up	Detection	Analytes	Matrices	Recovery (%) Range	LoQ Range	Validation Parameters Evaluated	Notes
Rùbies et al. (2015)	QuEChERS	UHPLC-ESI-Q-Orbitrap	AZA1, AZA2, AZA3, DTX1, DTX2, PTX1, PTX2, SPX1, OA, YTX, hYTX, 45OHYTX, 45OHhYTX	fresh and canned bivalve molluscs	69–119	25 µg kg^−1^	selectivity, linearity, trueness, precision	eprinomectin as internal standard
Blay et al. (2011)	SLE MeOH	LC-ESI-Orbitrap	AZA1, AZA2, AZA3, DTX1, DTX2, PTX2, PTX2, SPX1, OA, PSTs	shellfish	N/A	10–30 µg kg^−1^	linearity	screening
Regueiro et al. (2011)	SLE: MeOH/H_2_O online-SPE	HPLC-ESI-QqQ-MS/MS	AZA1, DTX1, DTX2, PTX2, SPX1, OA, YTX, GYM	mussels	97–102	1.12–8 µg kg^−1^	linearity, trueness, precision, matrix effect	
Fang et al. (2014)	SLE: MeOHSPE	UFLC-ESI-QqQ-MS/MS	AZA2, PTX2, SPX1, GYM	bivalve molluscs	71–101	0.037–0.27 µg kg^−1^	linearity, trueness, precision, matrix effect	
Rodríguez et al. (2018)	SLE: MeOH	HPLC-ESI-QqQ-MS/MS	AZA1, AZA2, AZA3, DTX1, DTX2, PTX2, SPX1, OA, YTX, hYTX, PSTs, TTX, DA	mussels	N/A	0.047–40.15 µg kg^−1^	linearity, precision, matrix effect	OA/DTX2 one peak; different extraction protocol for PSTs, TTX, DA
García-Altares et al. (2013)	SLE: MeOH	LC-QTRAP-ESI-MS/MS	AZA1, AZA2, AZA3, DTX1, DTX2, PTX2, SPX1, OA, YTX, hYTX, 45OHYTX, 45OHhYTX, GYM	bivalve molluscs	28–150	1.5–377 µg kg^−1^	linearity, precision, trueness, matrix effect	comparative study (different mobile phase pH)
These et al. (2009)	SLE: MeOH SPE	LC-QTRAP-ESI-MS/MS	AZA1, PTX2, OA, YTX,	bivalve molluscs and processed shellfish products	86–147	1 µg kg^−1^	linearity, trueness, precision	comparative study (different SPE cartridges)
Fux et al. (2009)	PEA	(1) HPLC-ESI-QqQ-MS/MS (2) HPLC-QTOF-MS/MS	AZA1, PTX2, OA	mussels	N/A	N/A	linearity, matrix effect	study of matrix effect evaluation
Wang et al. (2019)	QuEChERS dSPE	HPLC-ESI-QqQ-MS/MS	AZA1, AZA2, AZA3, DTX1, DTX2, SPX1, OA, YTX, hYTX	fresh and processed shellfish	88–109	0.32–4.92 µg kg^−1^	linearity, precision, trueness, matrix effect	comparative study (different sorbents)
Wang et al. (2015)	SLE: MeOH SPE	LC-QTRAP-ESI-MS/MS	DTX1, DTX2, PTX2, OA	bottlenose dolphin	85–140	0.2–0.7 µg kg^−1-^	linearity, precision, trueness	
Domènech et al. (2014)	SLE: MeOH	UHPLC-ESI-Q-Orbitrap	AZA1, PTX2, SPX1, OA, YTX, GYM	mussels	80–110	0.9–4.8 µg kg^−1^	selectivity, linearity, trueness, precision, measurement uncertainty	robust validation study
Schirone et al. (2018)	SLE: MeOH	HPLC-ESI-QqQ-MS/MS	AZA1, AZA2, AZA3, DTX1, DTX2, PTX2, OA, YTX, hYTX	mussels	85–104	40–60 µg kg^−1^	selectivity, linearity, trueness, precision, measurement uncertainty	monitoring study
Gerssen et al. (2009)	SLE: MeOH SPE	HPLC-ESI-QqQ-MS/MS	OA, YTX, AZA1, PTX2, GYM, SPX1	mussels, scallops and oysters	63–117	9 µg kg^−1^	linearity, trueness, precision, matrix effect	matrix effect study
Gerssen et al. (2010)	SLE: MeOH SPE	HPLC-ESI-QqQ-MS/MS	OA, YTX, AZA1, PTX2, SPX1	mussels, oysters, cockles and clams	102–111	16.4 µg kg^−1^	linearity, trueness, precision	comparative study (with/without SPE)
Van den Top et al. (2011)	SLE: MeOH SPE	HPLC-ESI-QqQ-MS/MS	AZA1, AZA2, AZA3, DTX1, DTX2, PTX2, OA, YTX, 45OHYTX	mussels, oysters and cockles	80–110	4–53 µg kg^−1^	linearity, trueness, precision, matrix effect	inter-laboratory validation study
Oller-Ruiz et al. (2021)	DLLME	HPLC-ESI-QqQ-MS/MS	AZA1, AZA2, AZA3, AZA4, AZA5, DTX1, DTX2, PTX2, SPX1, OA, GYM	seawater	82–123	0.7–19 ng L^−1^	linearity, trueness, precision, matrix effect	monitoring study
This method	SLE: MeOH SPE	HPLC-ESI-QqQ-MS/MS	AZA1, AZA2, AZA3, DTX1, DTX2, PTX2, OA, YTX, hYTX, 45OHYTX, 45OHhYTX	fresh and processed mussels, oysters, scallops, clams, cockles and cephalopod molluscs	73–101	3–8 µg kg^−1^	selectivity, linearity, trueness, precision, matrix effect, measurement uncertainty, ruggedness	

SLE: solid-liquid extraction; SPE: solid-phase extraction; dSPE: dispersive solid-phase extraction; QuEChERS: “quick, easy, cheap, effective, rugged, and safe” extraction; PEA: post- extraction addition; DLLME: dispersive liquid–liquid microextraction; H_2_O: water; MeOH: methanol.HPLC-ESI-QqQ-MS/MS: high performance liquid chromatography- electrospray ionization- triple quadrupole- tandem mass spectrometry; UFLC-ESI-QqQ-MS/MS: ultra-fast liquid chromatography- electrospray ionization- triple quadrupole- tandem mass spectrometry; UHPLC-ESI-Q-Orbitrap: ultra-high performance liquid chromatography- electrospray ionization- quadrupole- Orbitrap; LC-ESI-Q-TRAP: liquid chromatography- electrospray ionization- quadrupole- ionic trap, HPLC-QTOF-MS: high performance liquid chromatography—quadrupole -time of flight tandem mass spectrometry. AZA1: azaspiracid-1, AZA2 azaspiracid-2, AZA3 azaspiracid-3, AZA4 azaspiracid-4, AZA5 azaspiracid-5, DTX1: dinophysistoxin-1, DTX2: dinophysistoxin-2, PTX1: pectenotoxin-1, PTX2: pectenotoxin-2, SPX1: 13-desmethyl spirolide, OA: okadaic acid, YTX: yessotoxin, hYTX: homoyessotoxin, 45OHYTX: 45-hydroxy-yessotoxin, 45OHhYTX: 45-hydroxy-homoyessotoxin, GYM: gymnodimine, PSTs: paralytic shellfish toxins, TTX: tetrodotoxin, DA: domoic acid. N/A: not available.

**Table 7:** Proficiency test results.

Compounds	Assigned Values (x_a_) µg kg^−1^	Obtained Value µg kg^−1^	Obtained Z-Score
OA	302	382	1.20
Total OA	745	815	0.56
OA + PTX group	748	815	0.54
YTX	320	330	0.11
hYTX	2660	3550	2.05
YTX group	3940	3880	−0.11

## Supplementary Materials

The following are available online at https://www.mdpi.com/article/10.3390/md20030173/s1, Table S1: Concentration of lipophilic marine toxins in 1000 samples of shellfish from Italian market during the period 2019–2021. Table S2: Validation study: evaluation of matrix effect.

## References

[1] European Food Safety Authority (EFSA) (2008). Marine Biotoxins in Shellfish—Okadaic Acid and Analogues—Scientific Opinion of the Panel on Contaminants in the Food Chain. EFSA J..

[2] Gerssen A., Pol-Hofstad I.E., Poelman M., Mulder P.P., Van den Top H.J., De Boer J. (2010). Marine Toxins: Chemistry, Toxicity, Occurrence and Detection, with Special Reference to the Dutch Situation. Toxins.

[3] European Food Safety Authority (EFSA) (2009). Marine Biotoxins in Shellfish—Summary on Regulated Marine Biotoxins. EFSA J..

[4] Morabito S., Silvestro S., Faggio C. (2018). How the Marine Biotoxins Affect Human Health. Nat. Prod. Res..

[5] Gerssen A., Klijnstra M.D., Wong Y., Lewis R.J. (2017). The Determination of marine biotoxins in seafood. Analysis of Food Toxins and Toxicants.

[6] FAO—Food and Agriculture Organization of the United Nations, WHO—World Health Organization (2016). Technical Paper on Toxicity Equivalency Factors for Marine Biotoxins Associated with Bivalve Molluscs.

[7] Blanco J. (2018). Accumulation of Dinophysis Toxins in Bivalve Molluscs. Toxins.

[8] FAO—Food and Agriculture Organization of the United Nations (2004). Food and Nutrition Paper on Marine Biotoxins.

[9] Corriere M., Soliño L., Costa P.R. (2021). Effects of the Marine Biotoxins Okadaic Acid and Dinophysistoxins on Fish. J. Mar. Sci. Eng..

[10] Estevez P., Castro D., Pequeño-Valtierra A., Giraldez J., Gago-Martinez A. (2019). Emerging Marine Biotoxins in Seafood from European Coasts: Incidence and Analytical Challenges. Foods.

[11] Visciano P., Schirone M., Berti M., Milandri A., Tofalo R., Suzzi G. (2016). Marine Biotoxins: Occurrence, Toxicity, Regulatory Limits and Reference Methods. Front. Microbiol..

[12] European Food Safety Authority (EFSA) (2009). Marine Biotoxins in Shellfish—Yessotoxin Group-Scientific Opinion of the Panel on Contaminants in the Food Chain. EFSA J..

[13] European Food Safety Authority (EFSA) (2008). Marine Biotoxins in Shellfish—Azaspiracid Group-Scientific Opinion of the Panel on Contaminants in the Food Chain. EFSA J..

[14] European Food Safety Authority (EFSA) (2009). Marine Biotoxins in Shellfish—Pectenotoxin Group. EFSA J..

[15] O’Mahony M. (2018). EU Regulatory Risk Management of Marine Biotoxins in the Marine Bivalve Mollusc Food-Chain. Toxins.

[16] European Commission Regulation (EC). No 853/2004 of the European Parliament and of the Council of 29 April 2004 Laying down Specific Hygiene Rules for Food of Animal Origin. Official Journal of the European. Union 2004, L. 139/55. https://data.europa.eu/eli/reg/2004/853/2021-10-28.

[17] European Commission Regulation (EU). 627/2019 of 15 March 2019—Laying down Uniform Practical Arrangements for the Performance of Official Controls on Products of Animal Origin Intended for Human Consumption in Accordance with Regulation (EU) 625/2017 of the European Parliament and of the Council and Amending Commission Regulation (EC) No 2074/2005 as Regards Official Controls. Official Journal of the European Union 2019, L131/50. https://data.europa.eu/eli/reg_impl/2019/627/2021-10-14.

[18] Schirone M., Berti M., Visciano P., Chiumiento F., Migliorati G., Tofalo R., Suzzi G., Di Giacinto F., Ferri N. (2018). Determination of Lipophilic Marine Biotoxins in Mussels Harvested from the Adriatic Sea by LC-MS/MS. Front. Microbiol..

[19] Lee T., Fong F., Ho K.-C., Lee F. (2016). The Mechanism of Diarrhetic Shellfish Poisoning Toxin Production in Prorocentrum Spp.: Physiological and Molecular Perspectives. Toxins.

[20] Paz B., Daranas A., Norte M., Riobó P., Franco J., Fernández J. (2008). Yessotoxins, a Group of Marine Polyether Toxins: An Overview. Mar. Drugs.

[21] Alfonso A., Vieytes M., Botana L. (2016). Yessotoxin, a Promising Therapeutic Tool. Mar. Drugs.

[22] Ferreiro S.F., Vilariño N., Carrera C., Louzao M.C., Santamarina G., Cantalapiedra A.G., Cifuentes J.M., Vieira A.C., Botana L.M. (2017). Subacute Immunotoxicity of the Marine Phycotoxin Yessotoxin in Rats. Toxicon.

[23] Hu W., Xu J., Sinkkonen J., Wu J. (2010). Polyketides from Marine Dinoflagellates of the Genus Prorocentrum, Biosynthetic Origin and Bioactivity of Their Okadaic Acid Analogues. Mini Rev. Med. Chem..

[24] Valdiglesias V., Prego-Faraldo M., Pásaro E., Méndez J., Laffon B. (2013). Okadaic Acid: More than a Diarrheic Toxin. Mar. Drugs.

[25] Ferron P.-J., Dumazeau K., Beaulieu J.-F., Le Hégarat L., Fessard V. (2016). Combined Effects of Lipophilic Phycotoxins (Okadaic Acid, Azaspiracid-1 and Yessotoxin) on Human Intestinal Cells Models. Toxins.

[26] Toyofuku H. (2006). Joint FAO/WHO/IOC Activities to Provide Scientific Advice on Marine Biotoxins (Research Report). Mar. Pollut. Bull..

[27] European Commission Regulation (EU) 1374/2021 of 12 April 2021 Amending Annex III to Regulation (EC) No 853/2004 of the European Parliament and of the Council on Specific Hygiene Requirements for Food of Animal Origin. Official Journal of the European Union 2021, L 297/1. https://data.europa.eu/eli/reg_del/2021/1374/oj.

[28] Otero P., Rodríguez P., Botana A.M., Alfonso A., Botana L.M. (2013). Analysis of natural toxins. Liquid Chromatography.

[29] EU-RLMB—European Union Reference Laboratory for Marine Biotoxins (2015). EU-Harmonised Standard Operating Procedure for Determination of Lipophilic Marine Biotoxins in Molluscs by LC-MS/MS, Version 5. http://www.aesan.gob.es/AECOSAN/docs/documentos/laboratorios/LNRBM/ARCHIVO2EU-Harmonised-SOP-LIPO-LCMSMS_Version5.pdf.

[30] European Commission Commission Decision 657/2002 of 14 August 2002 Implementing Council Directive 96/23/EC Concerning the Performance of Analytical Methods and the Interpretation of Results. Official Journal of the European Union 2002, L 221/8. https://data.europa.eu/eli/dec/2002/657/2021-06-10.

[31] European Commission Regulation (EU) 2017/625 of the European Parliament and of the Council of 15 March 2017 on Official Controls and Other Official Activities Performed to Ensure the Application of Food and Feed Law, Rules on Animal Health and Welfare, Plant Health and Plant Protection Products, Amending Regulations (EC) No 999/2001, (EC) No 396/2005, (EC) No 1069/2009, (EC) No 1107/2009, (EU) No 1151/2012, (EU) No 652/2014, (EU) 2016/429 and (EU) 2016/2031 of the European Parliament and of the Council, Council Regulations (EC) No 1/2005 and (EC) No 1099/2009 and Council Directives 98/58/EC, 1999/74/EC, 2007/43/EC, 2008/119/EC and 2008/120/EC, and Repealing Regulations (EC) No 854/2004 and (EC) No 882/2004 of the European Parliament and of the Council, Council Directives 89/608/EEC, 89/662/EEC, 90/425/EEC, 91/496/EEC, 96/23/EC, 96/93/EC and 97/78/EC and Council Decision 92/438/EEC (Official Controls Regulation). Official Journal of the European Union 2017, L 95/1. https://data.europa.eu/eli/reg/2017/625/2021-10-28.

[32] ICH Expert Working Group (2006). ICH Harmonised Guideline. Validation of Analytical Procedures: Text and Methodology.

[33] EURACHEM (2014). Eurachem Guideline—The Fitness for Purpose of Analytical Methods. A Laboratory Guide to Method Validation and Related Topics.

[34] (2017). General Requirements for the Competence of Testing and Calibration Laboratories.

[35] (2019). Accuracy (Trueness and Precision) of Measurement Methods and Results—Part 2: Basic Method for the Determination of Repeatability and Reproducibility of a Standard Measurement Method.

[36] (2010). Conformity Assessment—General Requirements for Proficiency Testing.

[37] European Food Safety Authority (EFSA) (2010). Management of Left-Censored Data in Dietary Exposure Assessment of Chemical Substances. EFSA J..

[38] These A., Scholz J., Preiss-Weigert A. (2009). Sensitive Method for the Determination of Lipophilic Marine Biotoxins in Extracts of Mussels and Processed Shellfish by High-Performance Liquid Chromatography–Tandem Mass Spectrometry Based on Enrichment by Solid-Phase Extraction. J. Chromatogr. A.

[39] Wang L., Shi X., Zhao Q., Sun A., Li D., Zhao J. (2019). Determination of Lipophilic Marine Toxins in Fresh and Processed Shellfish Using Modified QuEChERS and Ultra-High-Performance Liquid Chromatography–Tandem Mass Spectrometry. Food Chem..

[40] Gerssen A., McElhinney M.A., Mulder P.P.J., Bire R., Hess P., de Boer J. (2009). Solid Phase Extraction for Removal of Matrix Effects in Lipophilic Marine Toxin Analysis by Liquid Chromatography-Tandem Mass Spectrometry. Anal. Bioanal. Chem..

[41] Fux E., Rode D., Bire R., Hess P. (2008). Approaches to the Evaluation of Matrix Effects in the Liquid Chromatography-Mass Spectrometry (LC-MS) Analysis of Three Regulated Lipophilic Toxin Groups in Mussel Matrix (*Mytilus edulis*). Food Addit. Contam. Part A.

[42] Fang L., Yao X., Wang L., Li J. (2015). Solid-Phase Extraction-Based Ultra-Sensitive Detection of Four Lipophilic Marine Biotoxins in Bivalves by High-Performance Liquid Chromatography-Tandem Mass Spectrometry. J. Chromatogr. Sci..

[43] Regueiro J., Rossignoli A.E., Álvarez G., Blanco J. (2011). Automated On-Line Solid-Phase Extraction Coupled to Liquid Chromatography–Tandem Mass Spectrometry for Determination of Lipophilic Marine Toxins in Shellfish. Food Chem..

[44] Gerssen A., Mulder P.P.J., McElhinney M.A., de Boer J. (2009). Liquid Chromatography–Tandem Mass Spectrometry Method for the Detection of Marine Lipophilic Toxins under Alkaline Conditions. J. Chromatogr. A.

[45] García-Altares M., Diogène J., de la Iglesia P. (2013). The Implementation of Liquid Chromatography Tandem Mass Spectrometry for the Official Control of Lipophilic Toxins in Seafood: Single-Laboratory Validation under Four Chromatographic Conditions. J. Chromatogr. A.

[46] Stobo L.A., Lacaze J.-P.C.L., Scott A.C., Gallacher S., Smith E.A., Quilliam M.A. (2005). Liquid Chromatography with Mass Spectrometry—Detection of Lipophilic Shellfish Toxins. J. AOAC Int..

[47] European Commission Commission Regulation (EU) No 15/2011 of 10 January 2011 Amending Regulation (EC) No 2074/2005 as Regards Recognised Testing Methods for Detecting Marine Biotoxins in Live Bivalve Molluscs. Official Journal of the European Union 2011, L 6/3. https://data.europa.eu/eli/reg/2011/15/oj.

[48] Rúbies A., Muñoz E., Gibert D., Cortés-Francisco N., Granados M., Caixach J., Centrich F. (2015). New Method for the Analysis of Lipophilic Marine Biotoxins in Fresh and Canned Bivalves by Liquid Chromatography Coupled to High Resolution Mass Spectrometry: A Quick, Easy, Cheap, Efficient, Rugged, Safe Approach. J. Chromatogr. A.

[49] Domènech A., Cortés-Francisco N., Palacios O., Franco J.M., Riobó P., Llerena J.J., Vichi S., Caixach J. (2014). Determination of Lipophilic Marine Toxins in Mussels. Quantification and Confirmation Criteria Using High Resolution Mass Spectrometry. J. Chromatogr. A.

[50] Blay P., Hui J.P.M., Chang J., Melanson J.E. (2011). Screening for Multiple Classes of Marine Biotoxins by Liquid Chromatography–High-Resolution Mass Spectrometry. Anal. Bioanal. Chem..

[51] Bacchiocchi S., Siracusa M., Ruzzi A., Gorbi S., Ercolessi M., Cosentino M.A., Ammazzalorso P., Orletti R. (2015). Two-Year Study of Lipophilic Marine Toxin Profile in Mussels of the North-Central Adriatic Sea: First Report of Azaspiracids in Mediterranean Seafood. Toxicon.

[52] Gerssen A., van Olst E.H.W., Mulder P.P.J., de Boer J. (2010). In-House Validation of a Liquid Chromatography Tandem Mass Spectrometry Method for the Analysis of Lipophilic Marine Toxins in Shellfish Using Matrix-Matched Calibration. Anal. Bioanal. Chem..

[53] Rodríguez I., Alfonso A., González-Jartín J.M., Vieytes M.R., Botana L.M. (2018). A Single Run UPLC-MS/MS Method for Detection of All EU-Regulated Marine Toxins. Talanta.

[54] Bosch-Orea C., Sanchís J., Farré M., Barceló D. (2017). Analysis of Lipophilic Marine Biotoxins by Liquid Chromatography Coupled with High-Resolution Mass Spectrometry in Seawater from the Catalan Coast. Anal. Bioanal. Chem..

[55] Oller-Ruiz A., Campillo N., Hernández-Córdoba M., Gilabert J., Viñas P. (2021). Monitoring Lipophilic Toxins in Seawater Using Dispersive Liquid—Liquid Microextraction and Liquid Chromatography with Triple Quadrupole Mass Spectrometry. Toxins.

[56] van den Top H.J., Gerssen A., McCarron P., van Egmond H.P. (2011). Quantitative Determination of Marine Lipophilic Toxins in Mussels, Oysters and Cockles Using Liquid Chromatography-Mass Spectrometry: Inter-Laboratory Validation Study. Food Addit. Contam. Part A.

[57] Wang Z., Broadwater M.H., Ramsdell J.S. (2015). Analysis of Diarrhetic Shellfish Poisoning Toxins and Pectenotoxin-2 in the Bottlenose Dolphin (Tursiops Truncatus) by Liquid Chromatography–Tandem Mass Spectrometry. J. Chromatogr. A.

[58] Gałuszka A., Migaszewski Z., Namieśnik J. (2013). The 12 Principles of Green Analytical Chemistry and the SIGNIFICANCE Mnemonic of Green Analytical Practices. TrAC Trends Anal. Chem..

